# Comprehensive Assessment of Visual Perceptual Skills in Autism Spectrum Disorder

**DOI:** 10.3389/fpsyg.2021.662808

**Published:** 2021-07-13

**Authors:** Antoinette Sabatino DiCriscio, Jaclyn Smith, Vanessa Troiani

**Affiliations:** ^1^Geisinger Health System, Autism and Developmental Medicine Institute (ADMI), Lewisburg, PA, United States; ^2^Department of Imaging Science and Innovation, Center for Health Research, Danville, PA, United States; ^3^Geisinger Neuroscience Institute, Danville, PA, United States; ^4^Department of Basic Sciences, Geisinger Commonwealth School of Medicine, Scranton, PA, United States

**Keywords:** visual perception, autism, individual differences, TVPS, global-local processing

## Abstract

The purpose of the current study was to assess meaningful variability in visual-perceptual skills using a standardized assessment of visual perception, the Test of Visual Perceptual Skills (TVPS), across children with and without autism spectrum disorder (ASD). In addition to assessing overall accuracy across subtests of the TVPS, we also assessed response variability at the item-level, and the linear relationship between quantitative measures of ASD symptoms, task performance, and item-level variance. We report a significant linear relationship between ASD features and performance on the TVPS Figure Ground subtest. Additionally, results of an item-level analysis point to a significant relationship between within-task variability on the Figure Ground subtest and quantitative ASD traits, with a less variable response pattern being associated with increased ASD symptoms. Findings presented here suggest variability in perceptual processing across ASD may be influenced by individual differences in trait distribution.

## Introduction

Visual perception has been researched at length in autism spectrum disorder (ASD). Several studies have demonstrated a perceptual bias to local as compared to global features and/or enhanced visual processing abilities in individuals with ASD (Happé, 1999; Dakin and Frith, 2005; see Simmons et al., 2009 for review). However, other studies have noted that enhanced perceptual performance in individuals with ASD may not be observed across all contexts (D'Souza et al., 2016; Guy et al., 2019). While some reports indicate poorer task performance on paradigms requiring global visual processing in individuals with ASD as compared to their peers, others have been unable to identify significant differences in perceptual precedence or global vs. local processing (Happé and Booth, 2008). Inconsistent results across the current literature have been attributed to variable sample demographics and the clinical characterization of diagnostic groups, variable task demands, differences in task administration, and differing stimulus properties (Van der Hallen et al., 2015). However, a large proportion of the current research utilizes specific perceptual paradigms designed to capture differences in global/local processing (i.e., Navon figures; EFT, Embedded figures test) and makes direct comparisons between those with ASD diagnoses and those that lack any diagnosis (Plaisted et al., 1998; O'Riordan et al., 2001; Dakin and Frith, 2005; Mottron et al., 2006; Scherf et al., 2008; Kaldy et al., 2011, 2016). Research in typically developing individuals or healthy adults suggests that there is also natural variability in perceptual precedence across the general population (Scherf et al., 2009; Dale and Arnell, 2010; McKone et al., 2010). Visual perception and perceptual performance in ASD thus may not be simply labeled as diminished or “impaired” global processing. Rather, meaningful variability in global-local processing across the autism spectrum may reflect a broader distribution of cognitive and perceptual strategies that also extends into the non-ASD population.

Existing work on visual perception and global-local processing in ASD has traditionally used research paradigms that require an individual to identify one of a finite set of smaller shapes within larger, more complex shapes (Witkin, 1971) or sort objects/stimuli according to local vs. global features. These tasks have been successful in identifying perceptual differences between a typical control group and those with ASD using summary scores and group average task performance metrics (i.e., accuracy or reaction time). However, some of the previously used paradigms may lack the precision necessary to relate individual differences in performance to ASD traits. Furthermore, previous studies on visual perception in ASD more often make use of a singular experimental paradigm or a restricted perceptual battery (i.e., Embedded Figures, Navon figures, or Block Design; Mottron et al., 2003; Stewart et al., 2009; Almeida et al., 2010; Horlin et al., 2014; Cribb et al., 2016) and rarely assess a range of visual perceptual skills that influence task performance on such paradigms. Visual perception represents a complex cognitive construct composed of subskills ranging from basic to more advanced), with composite skills working synergistically to integrate visual input (Ritter and Ysseldyke, 1976; Warren, 1993; Cate and Richards, 2000; Ayhan et al., 2015). It remains unclear how individual differences in visual perceptual skills, assessed via standardized assessment, may be associated with ASD traits and whether or not individuals with ASD demonstrate enhanced performance on standardized assessments of perception.

Few studies to date have dimensionally assessed individual differences in perceptual performance in children with neurodevelopmental differences with *and* without ASD. Our own recent research has demonstrated significant relationships between quantitative measures of ASD traits and performance on specific subtests of the Test of Visual Perceptual Skills (TVPS), a standardized assessment of visual perceptual skill that includes seven subtests that assess various aspects of perception (DiCriscio and Troiani, 2017, 2018). In a cohort of healthy adults, individuals with higher ASD trait load demonstrated increased performance on the Figure Ground subtest of the TVPS, which assesses an individual's ability to disembed a smaller figure from a larger shape (DiCriscio and Troiani, 2017). We also found that the relationship between ASD features and TVPS performance was specific to the Figure Ground subtest in a heterogeneous pediatric cohort of *N* = 54 (*n* = 17 with a clinical diagnosis of ASD) children with and without ASD (DiCriscio and Troiani, 2018). While our previous research highlights a relationship between ASD traits and TVPS Figure Ground performance, replication with a larger ASD cohort is necessary.

Standardized performance summary scores do not always capture meaningful with-in task variability and item-level response patterns across individuals, which may be associated with typical and atypical development (VanMeter et al., 1997). Previous studies have highlighted the quantitative assessment of task performance at the item-level within cognitive and psychometric assessments (Hallenbeck et al., 1965) and found significant relationships between item-level response variability and quantitative ASD traits (Hare-Harris et al., 2019). Items within each TVPS subtest are organized according to difficulty, becoming more difficult as the individual progresses through each item. Individuals are expected, based on the hierarchical item structure, to sequentially answer earlier (easier) items correctly and later (more difficult) items incorrectly, eventually reaching maximum ability or ceiling after a consecutive number of incorrect responses. Variability in correct and incorrect responses can be anticipated, especially around items that are approximate estimates of developmental ability; however, research in ASD has reported qualitatively atypical and non-sequential development in language and cognitive domains (Capute and Accardo, 1996; Eigsti et al., 2007, 2011) and on standardized assessments of cognitive processes (Hare-Harris et al., 2019). However, to our knowledge, there are no studies quantitatively characterizing item-level response in the context of visual perception and assessing the relationship between within-task variability and ASD traits.

The overall objective of the current research was to confirm replication of our previous findings of a relationship between ASD traits and TVPS Figure Ground performance in an independent and larger sample of children with ASD. We assessed children with ASD as well as children with neurodevelopmental concerns but no ASD diagnosis on all subtests of the Test of Visual Perceptual Skills-3rd edition (TVPS-3). Quantitative ASD traits were based on parent/caregiver report on the Broader Autism Phenotype Questionnaire (BAP-Q) (Hurley et al., 2007) and the Social Responsiveness Scales-2nd edition (SRS-2; Constantino and Todd, 2003; Constantino et al., 2003; Constantino and Gruber, 2012; Frazier et al., 2014). Based on our previous research and other studies that have found enhanced visual perceptual skill in ASD (Happé, 1996; Bertone et al., 2005; Almeida et al., 2010; Horlin et al., 2014), we predicted that BAP-Q scores would be a significant predictor of performance on the TVPS Figure Ground subtest, thus serving as a replication of our previous findings. Additionally, we explored the relationship between within-task variability and item-level responses on the TVPS and quantitative ASD traits using a novel index of response dispersion (Hare-Harris et al., 2019). We predicted that within-task variability and item-level response profiles on the TVPS would be significantly associated with ASD traits.

## Methods

### Participants

We used a broad recruitment strategy in a real-world clinic setting in order to obtain a cohort with a wide range of ASD traits. Children are seen at Geisinger's Autism & Developmental Medicine Institute for a variety of reasons, including follow-up due to high risk for atypical neurodevelopment (premature birth), referral from primary care due to clinical suspicion of a neurodevelopmental condition, and/or parental concern and referral. All families seen at our clinic are approached to consent into a clinic-research protocol, which allows for recontact and additional research participation, such as in the study described here. Thus, this recruitment strategy results in a sample that is enriched for children with a range of neurodevelopmental concerns, including ASD, other neurodevelopmental diagnoses (i.e., not an ASD diagnosis), subclinical neurodevelopmental traits, as well as those that would be characterized as typically developing. Thus, this sample does not represent a more traditional matched clinical-control group, but includes those that have ASD diagnoses, those with other neurodevelopmental disorders, and children that are typically developing. We analyze the overall group dimensionally, as well as split into ASD and non-ASD subgroups.

Eighty-seven children, ages 5–16 years, participated in this study (mean age = 8.64 ± 2.33, 54 males). Forty-eight children of our larger sample of *N* = 87 had a diagnosis of ASD (ASD subsample: mean age = 8.77 ± 2.33, 41 males). Thirty-nine (*n* = 39) participants within our larger sample were identified and defined based on not having an ASD diagnosis. Some of these non-ASD participants did have other diagnoses or a reported history of neurodevelopmental diagnosis, including attention deficit hyperactivity disorder (ADHD; *n* = 6), language disorder (*n* = 4), a mood or emotional disorder (*n* = 3), minor coordination disorder (*n* = 1), and/or behavioral disorder (*n* = 1). Other children could be considered “typical,” lacking any clinical neurodevelopmental diagnosis.

We identified participants with a confirmed clinical diagnosis of autism or ASD, and/or autism symptoms that reached the critical threshold for an ASD diagnosis, conferred by our clinic's team of ASD experts, including neurodevelopmental pediatricians and clinical psychologists. The vast majority of patients (> 85%) receiving clinical care at our neurodevelopmental pediatric clinic consent/assent to a clinic-wide research protocol, which gives permission to access the patient's health record and allows for recontact for additional research. ASD and comorbid diagnoses for this study were determined based on a DSM-5 clinical diagnosis from a diagnostic team at Geisinger's Autism & Developmental Medicine Institute. After receiving a referral to our neurodevelopment clinic, patients undergo assessment by a multi-disciplinary team that includes neurodevelopment pediatricians, clinical psychologists, behavioral specialists, and speech pathologists. The clinical team may sometimes utilize an assessment tool such as the ADOS or ADI-R, but diagnoses are ultimately made by the clinicians using DSM-5 criteria for ASD after a comprehensive evaluation of the patient. ADOS and ADI-R scores were not available from the electronic health record for enough patients in the current sample and thus were not used in any analyses. All patient diagnoses, including ASD and any comorbidities, are entered into the patient's digital health record and available for this study. Due to the broad variety of patients seen in our clinic, we initially screened possible participant's health records to identify patients with an approximate IQ of 60 and higher and those not deemed non-verbal, or unable to provide simple verbal and/or manual responses, use small phrases, and understand simple commands were not recruited into the study. All participants consented to a research protocol approved by the institutional review board (IRB) at the authors' home institution. Sample demographics for the entire sample (*N* = 87) as well as the ASD subsample (*n* = 48) and non-ASD subsample (*n* = 39) are reported in Table 1.

**Table 1:** Sample demographics, BAP-Q, and SRS scores for entire sample and ASD and non-ASD subsamples.

	**Total sample (*****N*** **= 87)**			***ASD*** **(*****n*** **= 48 of 87)**			***Non-ASD*** **(*****n*** **= 39 of 87)**			p*
**Sex (M:F)**	**54:33**			**41:7**			**13:26**			^**^
	**Mean (St Dev)**	**Min**	**Max**	**Mean (St Dev)**	**Min**	**Max**	**Mean (St dev)**	**Min**	**Max**	
Age	8.64 (2.33)	5	16	8.77 (2.33)	6	16	8.55 (2.45)	5	13	--
FSIQ	97.13 (18.30)	46	131	90.08 (19.46)	46	131	106.53 (11.95)	84	128	^**^
BAP-Q Total	3.37 (0.98)	1.33	5.72	3.92 (0.87)	1.33	5.72	2.65 (0.61)	1.53	4.61	^**^
Aloof	3.09 (1.09)	1.17	5.25	3.50 (1.07)	1.17	5.25	2.51 (0.84)	1.25	4.50	^**^
Pragmatic language	3.28 (1.04)	1.58	6.00	3.88 (0.91)	1.75	6.00	2.5 (0.60)	1.58	4.00	^**^
Rigidity	3.69 (1.65)	1.08	6.00	4.31 (1.03)	1.08	6.00	2.78 (0.71)	1.75	5.58	^**^
SRS-2 Total (raw score)	67.10 (40.41)	3	154	87.75 (36.02)	16	154	39.34 (27.48)	3	107	^**^
SCI	54.94 (32.43)	3	122	70.69 (29.88)	12	122	33.71 (22.43)	3	88	^**^
RBRI	12.16 (8.80)	0	34	17.01 (7.23)	0	34	5.63 (5.90)	0	24	^**^
Social awareness	9.52 (4.57)	0	20	11.40 (4.67)	2	20	7.00 (3.12)	0	13	^**^
Social cognition	12.63 (7.91)	0	28	16.40 (7.10)	1	28	7.58 (5.85)	0	20	^**^
Social communication	21.90 (14.02)	1	55	28.65 (12.84)	1	55	12.74 (9.86)	1	40	^**^
Social motivation	10.97 (7.57)	0	29	14.25 (7.46)	0	29	14.39 (7.30)	0	29	^**^

-- *p>0.05, NS;*

** *p < 0.001*.

### Behavioral Measures and Scoring

#### Test of Visual Perceptual Skills-3rd Edition (TVPS-3)

The TVPS-3 is a standardized measure of an individual's (ages 4 and older) perceptual abilities. The test uses black and white line designs as stimuli across each of the seven subtests. Each item is administered in a multiple-choice format. The seven subtests, each containing 2 example items and 16 test items, are administered in succession, in the order below. Simple verbal responses are required from each participant making this perceptual assessment appropriate for children with a range of abilities including neurodevelopmental diagnoses (Wan et al., 2015). Due to copyright constraints, we are unable to provide figures illustrating all of the TVPS subtests.

**Figure 1:**
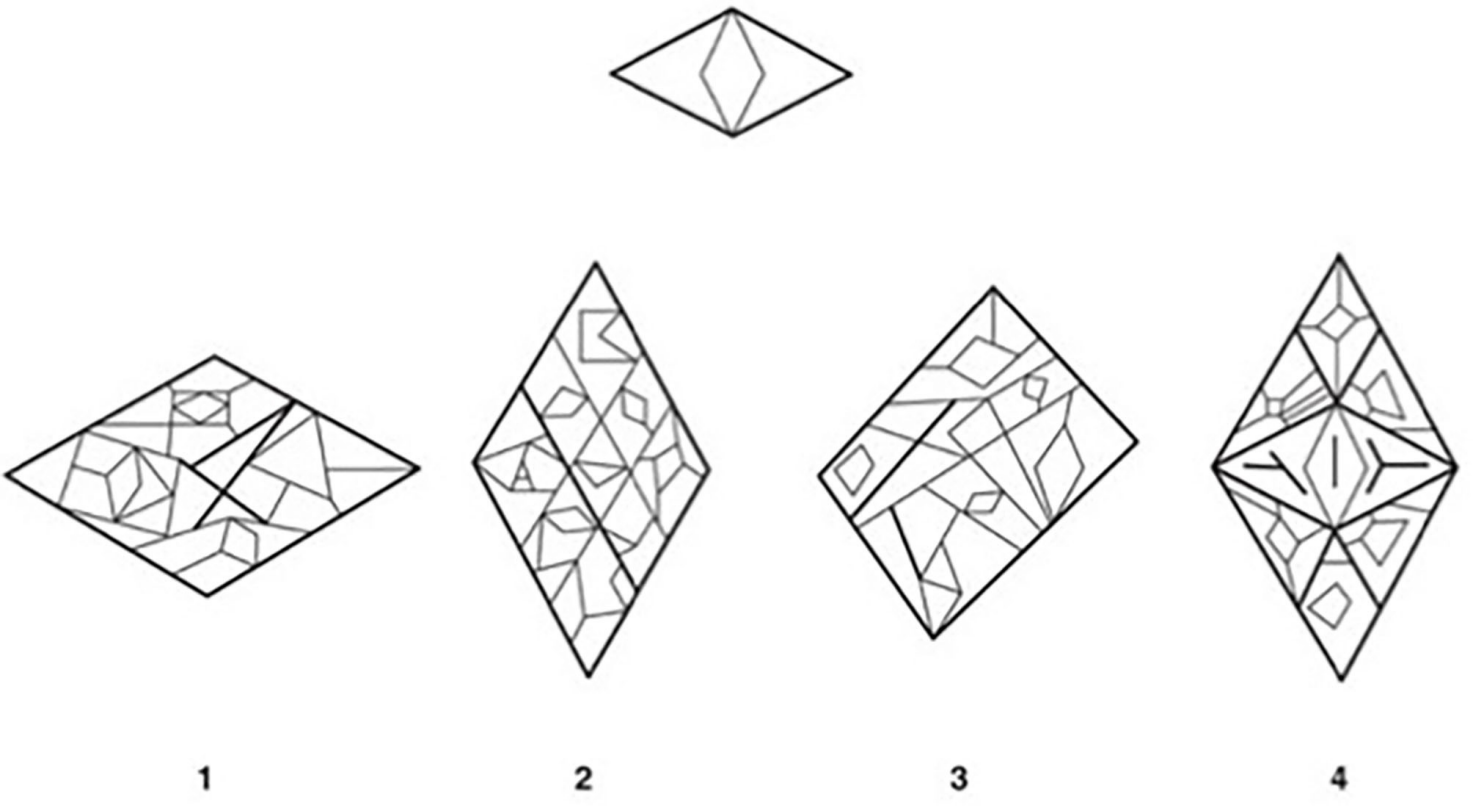
Example item from the Figure-Ground subtest of the TVPS-3. Participants are asked to find a simple image, or a design (top) nested within one of four more complex shapes (bottom). Correct answer in the above example: 4. The example TVPS-3 item illustrated above (Martin and Gardner, 2006) has been reproduced with permission from Academic Therapy Publications.

1. Visual Discrimination (VD): the individual is shown a picture or design and asked to identify the matching design at the bottom of the page.

2. Visual Memory (VM): the individual is shown a picture or design for 5 s, the page is turned, and the child is asked to identify the matching design on the new page.

3. Spatial Relationships (SR): the individual is shown a series of pictures or designs and asked to identify the one that is different, they are advised that it “may differ in detail or in the rotation of all or part of the design.”

4. Form Constancy (FC): the individual is asked to identify one picture or design on the page, it can be larger, smaller, or rotated.

5. Sequential Memory (SM): the individual is shown an arrangement of pictures or designs for 5 s and then asked to identify the matching design on the next page. The number of items in the arrangement increases throughout the test.

6. Figure-Ground (FG): the individual is asked to identify an image or design within a more complex shape. See Figure 1 for sample item.

7. Visual Closure (VC): the individual is shown a completed picture or design and asked to match it to an incomplete version on the page.

#### TVPS-3 Scoring and Performance Measures

Each correct response is scored as “1” and a total score is recorded at the end of each subtest. Raw scores are then converted to scaled scores (0–19) and percent ranks. The overall score and index scores are calculated from the sum of the scaled scores and conveyed as standard scores.

Group average TVPS index, subtest scores, and percentile ranks for children with ASD are described in Table 2. Group average TVPS-Overall scores in our ASD cohort (mean = 95.40 ± 15.48) were within average range. Performance in our ASD participants was comparable to TVPS Overall scores in a validity sample of *n* = 14 children with ASD, ages 5–16 years, outlined in the TVPS manual (mean = 90.54 ± 16.17) and fell within one standard deviation of the expected mean. To our knowledge, this is the largest study to date reporting TVPS performance in an ASD cohort.

**Table 2:** Group average TVPS scores, percentile ranks, and age equivalents within ASD sample (*n* = 48).

	**ASD subsample (*****n*** **= 48 of 87)**				**Age equivalents (TVPS-3)**		
	**Mean (St Dev)**	**Min**	**Max**	**% Ranks**	**Mean (St Dev)**	**Min**	**Max**
**TVPS index scores—standard scores**							
Overall	95.40 (15.84)	63	136	37	7.89 (3.34)	3.92	17.67
Basic processing	95.10 (16.71)	61	137	37	7.75 (3.29)	3.92	18.75
Sequencing	94.38 (16.71)	55	125	34	8.11 (3.90)	3.92	19.00
Complex processes	97.88 (18.65)	62	145	45	8.44 (4.23)	3.92	19.00
**TVPS subtest scores—scaled scores**							
Visual discrimination	8.69 (3.60)	0	16	37	7.63 (3.01)	3.92	14.75
Visual memory	8.31 (3.84)	0	18	25	7.46 (3.41)	3.92	19.00
Spatial relations	10.33 (5.20)	0	19	50	9.65 (5.00)	3.92	19.00
Form constancy	8.63 (4.64)	0	19	37	7.91 (4.38)	3.92	19.00
Sequential memory	8.83 (3.45)	0	15	37	8.14 (3.88)	3.92	19.00
Figure-ground	10.42 (4.43)	2	19	50	9.71 (5.19)	3.92	19.00
Visual closure	8.65 (3.81)	0	19	37	7.64 (3.88)	3.92	19.00

*Group average percentile ranks for index standard and subtest scaled scores were calculated based on scoring procedures outlined in the TVPS manual. Reported percentile ranks reflect group average performance relative to the normative population. TVPS index and overall scores are reported as standard scores based on population distribution with a mean of 100 and standard deviation of 15*.

#### TVPS-3 Response Dispersion Index

A modified Response Dispersion Index (RDI) score across each of the TVPS subtests was calculated based on previously published methods (Hallenbeck et al., 1965; VanMeter et al., 1997; Hare-Harris et al., 2019). RDI has been theoretically interpreted as an inefficiency metric and index of within-task variability at the item-level (VanMeter). This modified response index accounts for increasing item difficulty within each subtest. Given the ceiling/stop rule (i.e., 3 consecutive incorrect responses), items that were not administered due to the individual reaching ceiling were coded as incorrect responses. To quantify the variance of responses for each individual, a modified RDI was calculated as the sum of the weight of the items missed. As each subtest of the TVPS has 16 items with increasing difficulty, the weight of each item was calculated starting at 1 and decreased by 1/16th (Hare-Harris et al., 2019). Possible scenarios which influence a higher RDI score include (i) answering easier items incorrectly while answering harder items correctly or (ii) increased failure on individual items after long runs of correct responses. Thus, higher RDI scores indicate a more variable and/or inefficient response pattern. See Table 3 for example RDI calculation that demonstrates variability in RDI across individuals with the same subtest accuracy score. Subj #001 responds to items sequentially, providing more correct responses earlier in test administration before reaching “ceiling” and stopping test administration. Subj #002 also responds to early items sequentially; however, appears to demonstrate a more variable response pattern and provides more incorrect responses to earlier test items while getting later (possibly more difficult) items correct and not reaching “ceiling”. RDI scores were calculated separately for each TVPS subtest (i.e., resulting in seven RDI subtest scores) and used in analysis focusing on (i) differences in item-level-response accuracy between participants with and without ASD and (ii) individual differences in item-level accuracy associated with quantitative ASD traits.

**Table 3:** Response dispersion index schematic.

	**Increasing Item Difficulty → → → →**																	
**Item #**	**1**	**2**	**3**	**4**	**5**	**6**	**7**	**8**	**9**	**10**	**11**	**12**	**13**	**14**	**15**	**16**	**Σ**	**RDI**
*Item weight*	*1*	*0.9375*	*0.875*	*0.8125*	*0.75*	*0.6875*	*0.625*	*0.5625*	*0.5*	*0.4375*	*0.375*	*0.3125*	*0.25*	*0.1875*	*0.125*	*0.0625*		
Subj #001	1	1	1	1	1	1	1	0	0	0	0	0	0	0	0	0	7	2.8125
Subj #002	1	1	1	0	0	1	0	0	1	0	0	1	0	0	1	0	7	4.0625

#### Broader Autism Phenotype Questionnaire

The Broader Autism Phenotype Questionnaire (BAP-Q) (Hurley et al., 2007) is a questionnaire designed to assess quantitative features across core symptom domains of ASD (Social Aloof, Rigidity, Pragmatic Language). The BAP-Q was created based on clinical assessment of parents with a child with an ASD diagnosis and is often completed via a self-report form in adults. However, previous research has successfully utilized the BAP-Q to assess quantitative ASD traits across children with and without ASD (DiCriscio and Troiani, 2017). Parents were asked to rate how often statements refer to their child, using a 6-point Likert scale ranging from very rarely to very often. Scores within each subscale are averaged. The average of the subscale scores is combined to create an overall average total score, reflecting overall ASD trait load across each of the three subscales. Total average and average subscale scores for this sample can be found in Table 1.

#### The Social Responsiveness Scale-2nd Edition

The Social Responsiveness Scale-2nd Edition (SRS-2; Constantino et al., 2003; Frazier et al., 2014), is most commonly used as a parent-report measure assessing the presence and severity of symptoms of social impairments associated with ASD. In addition to a Total score of overall impairment and a score of Social Communication Impairment (SCI), scores are calculated across five subscales (Restricted Interests and Repetitive Behavior, Social Awareness, Social Cognition, Social Communication, and Social Motivation). Scores can be converted to standardized t-scores that indicate symptom severity based upon a provided range; however, in order to maximize phenotypic variability in the current sample, raw SRS scores were used in all analyses described below.

We wish to emphasize that our interest in individual differences in visual perceptual features and ASD traits across our pediatric cohort underscored the use of questionnaires and behavioral assessments described above (i.e., the BAP-Q and the SRS). While the BAP-Q has been more consistently used in adult samples, previous work has demonstrated significant relationships between parental BAP-Q and child SRS scores (Maxwell et al., 2013). Given that there is not yet widespread use of the BAP-Q in neurodevelopment populations described in the current context, both measures were implemented in order to test our primary hypothesis of the relationship between BAP-Q and TVPS scores based on previous studies (DiCriscio and Troiani, 2017, 2018). We utilized measures that aligned with a quantitative approach and the dimensional assessment of perceptual skills and ASD traits as opposed to more definitive diagnostic measures that are designed for capturing impairment in those with autism and do not emphasize the capture of meaningful variability across individuals with and without a clinical diagnosis.

### Data Analysis

#### Assessing Normality and Relationships Among Behavioral Measures

Prior to completing our formal analyses, we assessed the distribution of the data with a Shapiro-Wilks test of normality. We also explored the presence of any significant relationships between our behavioral measures and demographic variables (i.e., age and FSIQ) via pairwise correlations. The complete outcome of these tests can be found in Supplement 1. Overall, analyses indicated non-normal distributions as well as significant relationships between parental report measures (BAP-Q, SRS) and FSIQ. Based on the outcome of these analyses, our planned comparisons (described below) were completed using non-parametric tests and statistical tests that account for multicollinearity.

#### TVPS Performance in ASD vs. Non-ASD

Although the primary focus of the current research was to replicate and extend previous findings demonstrating a link between the dimensional assessment of autism traits and performance on the TVPS-FG subtest, we first assessed possible differences in TVPS performance between children with and without ASD across all TVPS index and subtest scores. Given that not all TVPS subtest scores demonstrated a normal distribution, a non-parametric, Mann-Whitney *U* test was used to assess between groups differences in TVPS performance between ASD and non-ASD subsamples.

#### Individual Differences on TVPS Performance and Quantitative ASD Traits

Based on previous work, we hypothesized a linear relationship between ASD features as measured via the BAP-Q and TVPS-FG subtest scores (DiCriscio and Troiani, 2017, 2018). Mean-centered FSIQ and mean-centered behavioral measures were included in linear regression models to account for multicollinearity as well any relationship driven by differences in IQ. A stepwise linear regression using Age, FSIQ, BAP-Q Total Average, and SRS Total raw score as predictors of TVPS-FG scores was run in order to quantify the relationship between TVPS-FG and ASD features.

#### TVPS-RDI in ASD vs. Non-ASD

We also explored possible differences in TVPS item-level response variability using RDI scores that were calculated across each of the TVPS subtests. Given that not all TVPS RDI subtest scores demonstrated a normal distribution, a non-parametric, Mann-Whitney *U*-test was used to quantify group differences in TVPS RDI scores between ASD and non-ASD subsamples.

#### Individual Differences in TVPS-RDI and Quantitative ASD Traits

To our knowledge, no study to date has assessed the utility of item-level response dispersion or response variability within the context of visual perception as it relates to autism traits. To quantify item-level response variability, we calculated a modified RDI score (see above). Regression analyses were used to explore the linear relationship between autism traits and TVPS-FG RDI scores. Finally, partial correlation with correction, including age and FSIQ as covariates, were used to assess the relationship between TVPS RDI subtest scores and ASD traits.

#### Item-Level Performance Across Groups With “High” and “Low” BAP Features

Other work examining differences in visual perception in ASD (and within the “typical” population) frequently groups individuals according to their scores on a questionnaire assessing autism traits (Cribb et al., 2016). Here, we grouped participants based on having “high” BAP features (i.e., above the median) and “low” BAP features, based on BAP-Q Total scores. We then assessed the differences in the proportion of correct responses for each item of the TVPS-FG subtest between groups of participants with “high” or “low” BAP features using chi-square tests of independence.

### Supplemental Analyses

In addition to our planned analyses described above, we completed a number of supplemental analyses to further understand interactions between measured trait dimensions and their impact on these results. These include differences between groups on TVPS-FG measures of accuracy and item response variability, with group definitions based on “high” and “low” BAP-Q. Finally, to better understand the impact of differences in FSIQ, we did a similar supplemental analysis, but differentiated groups based on a median split of FSIQ scores.

## Results

### TVPS Performance in ASD vs. Non-ASD

**Figure 2:**
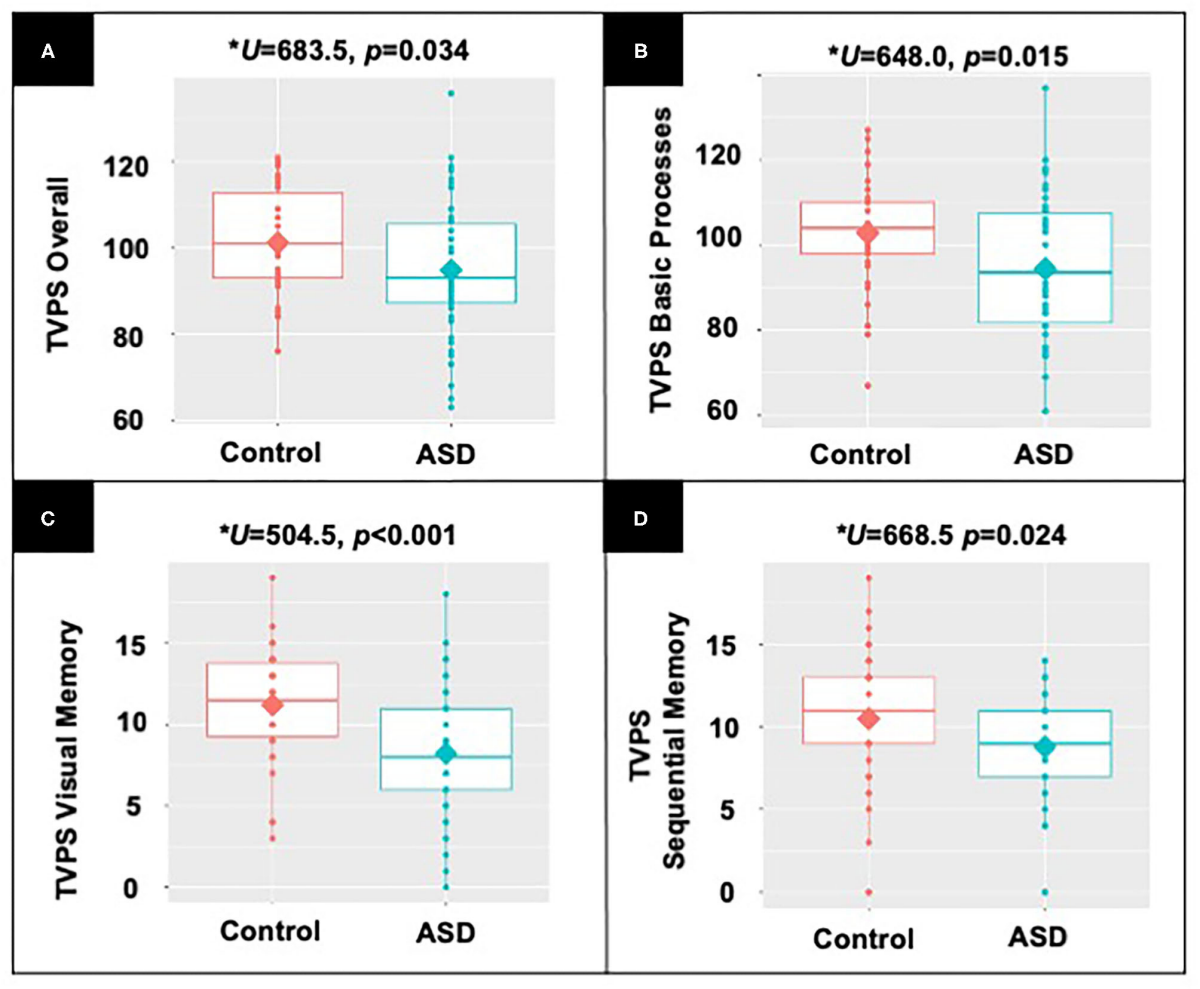
Results from non-parametric, Mann-Whitney *U*-demonstrating differences between participants with and without ASD in **(A)** TVPS Overall index scores; **(B)** TVPS Basic Processes subtest scores; **(C)** TVPS-VM subtest scores; and **(D)** TVPS-SM subtest scores.

Children without ASD demonstrated significantly higher TVPS Overall scores (*U* = 683.5, *p* = 0.034), TVPS Basic Processes scores (*U* = 648.0, *p* = 0.015), as well as TVPS Visual Memory (*U* = 504.5, *p* < 0.001) and Sequential Memory (*U* = 668.5, *p* = 0.024) subtest scores as compared to children with ASD (see Figure 2). Children with and without ASD did not differ on TVPS Sequencing (*U* = 701.5, *p* = 0.128, NS) and TVPS Complex Processes scores (*U* = 923.00, *p* = 0.945, NS) nor remaining TVPS subtest scores (*p*'s > 0.06, NS). Complete results are reported in Supplements 2, 3.

### Individual Differences on TVPS Performance and Quantitative ASD Traits

A regression equation including BAP-Q Total Average as well as FSIQ was significant [*F*_(2, 86)_ = 16.18, *p* < 0.001, *R*^2^ = 0.28, Adj *R*^2^ = 0.28]. Both FSIQ and BAP-Q Total Average were significant predictors of TVPS-FG scores (*p*'s < 0.003). SRS Total raw score as well as age were not found to be significant predictors of TVPS-FG (*p*'s < 0.88, NS). These results align with previous studies (DiCriscio and Troiani, 2017, 2018) and demonstrate that TVPS-FG scores can be predicted by ASD features as measured by the BAP-Q above and beyond the contribution of FSIQ.

**Figure 3:**
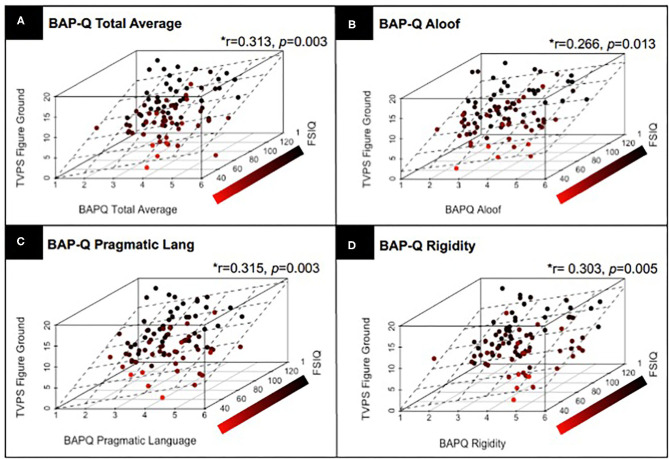
Results from a partial correlation (FSIQ) with correction for multiple comparisons, across children with and without ASD, demonstrating the relationship between TVPS-FG subtest scores and **(A)** BAP-Q Total scores; **(B)** BAP-Q Aloof subscales scores; **(C)** BAP-Q Pragmatic Language Scores; and **(D)** BAP-Q Rigidity subscale scores. Linear planes represent the results of a linear regression using FSIQ and **(A)** BAP-Q Total scores as well as **(B–D)** BAP-Q Aloof, Pragmatic Language, and Rigidity subscale scores to predict TVPS-FG subtest scores across children with and without ASD.

Next, we examined whether there is a relationship between ASD traits assessed via the BAP-Q and individual differences in visual perceptual skill using the TVPS via partial correlation, controlling for FSIQ and with correction for multiple comparisons. Results indicated a significant relationship between TVPS-FG and BAP-Q Total average score (*r* = 0.313, *p* = 0.003), BAP-Q Aloof (*r* = 0.266, *p* = 0.013), BAP-Q Pragmatic Language (*r* = 0.315, *p* = 0.003), as well as BAP-Q Rigidity (*r* = 0.303, *p* = 0.005). Thus, participants with higher ASD traits had higher scores on the TVPS-FG subtest (see Figures 3A–D). There were no other significant relationships with the other six TVPS subtest scores and BAP-Q scores (*p*'s > 0.13, NS). See Table 4 for complete results. These results underscore findings from previous research that have reported significant relationships between quantitative measures of ASD traits and visual perceptual skills specific to the TVPS-FG subtest. Given the broad age range of our sample, we repeated analyses, including both age and FSIQ as covariates. Results were identical to those reported above. See Supplement 4.

**Table 4:** Partial correlation (FSIQ) between BAP-Q and TVPS scores with correction for multiple comparisons.

		**BAP-Q**				**TVPS subtests**						
		**Total** **average**	**Aloof**	**Prag lang**	**Rigidity**	**VD**	**VM**	**SR**	**FC**	**SM**	**FG**	**VC**
BAP-Q	Total Average	1.00 –										
	Aloof	0.847^**^ **<0.001**	1.00 –									
	Prag Lang	0.839^**^ **<0.001**	0.575^**^ **<0.001**	1.00 –								
	Rigidity	0.895^**^ **<0.001**	0.700^**^ **<0.001**	0.686^**^ **<0.001**	1.00 –							
TVPS subtests	VD	0.125 0.251	0.092 0.398	0.140 0.198	0.138 0.206	1.00 –						
	VM	−0.099 0.364	−0.039 0.719	−0.085 0.437	−0.165 0.129	0.330^**^ **0.002**	1.00 –					
	SR	0.045 0.683	0.054 0.620	0.068 0.533	−0.002 0.983	0.331^**^ **0.002**	0.200 0.065	1.00 –				
	FC	0.099 0.365	−0.008 0.974	0.138 0.204	0.141 0.197	0.312^**^ **0.003**	0.171 0.116	0.163 0.133	1.00 –			
	SM	−0.026 0.811	−0.004 0.974	0.078 0.478	−0.080 0.466	0.188 0.083	0.220^*^ 0.042	0.317^**^ **0.003**	0.252^*^ **0.019**	1.00 –		
	FG	0.313^**^ **0.003**	0.266^*^ **0.013**	0.315^**^ **0.003**	0.303^**^ **0.005**	0.520^**^ **<0.001**	0.262^*^ **0.015**	0.337^**^ **0.002**	0.313^**^ **0.003**	0.389^**^ **<0.001**	1.00 –	
	VC	0.041 0.708	0.059 0.589	0.110 0.312	−0.029 0.792	0.464^**^ **<0.001**	0.061 0.578	0.181 0.095	0.215^*^ **0.046**	0.321^**^ **0.003**	0.476^**^ **<0.001**	1.00 –

* *p < 0.05;*

** *p < 0.01. Bolded values are highlight significant results*.

Given that a majority of our ASD subsample was male, we explored possible group differences in TVPS performance between males with ASD as compared to those without ASD. Male participants with and without ASD did not demonstrate differences in TVPS performance across TVPS subtests (*p*'s > 0.082, NS), with the exception of the TVPS-SM subtest (*p* = 0.014). Male participants without ASD scored higher on the TVPS-SM subtest (mean = 11.79 ± 3.64) as compared to male participants with ASD (mean = 8.78 ± 3.13). We also repeated correlation analyses (described above) characterizing the relationship between ASD traits and TVPS performance and included only males with an ASD diagnosis (*n* = 41). Results with only male ASD participants confirm the relationship between autism traits and TVPS-FG scores. See Supplement 5 for results. These findings align with our previously published work reporting a specific relationship between performance on the TVPS-FG and ASD traits as measured by the BAP-Q Aloof subscale in healthy adult males (DiCriscio and Troiani, 2017).

### TVPS-RDI in ASD vs. Non-ASD

RDI scores across TVPS subtests were next compared between participants with and without ASD. TVPS Visual Memory RDI scores in children with ASD (mean = 2.792) as compared to non-ASD (mean = 2.064) approached but did not reach significance (*p* = 0.053, NS). No other differences in TVPS subtest RDI scores were noted (*p*'s > 0.375). See Table 5 for a summary TVPS RDI scores across each of the subtests.

**Table 5:** TVPS RDI scores across each of the seven TVPS subtests.

	**Total (*****n*** **= 87)**			**ASD subsample (*****n*** **= 48)**		
	**$\bar{x}(\sigma_{\bar{x}})$**	**Min**	**Max**	**$\bar{x}(\sigma_{\bar{x}})$**	**Min**	**Max**
**TVPS RDI**						
Visual discrimination	4.27 (1.46)	0	8.50	4.21 (1.50)	0	8.50
Visual memory	2.47 (1.69)	0	7.50	2.79 (1.75)	0	7.50
Spatial relations	2.22 (2.09)	0	7.50	2.46 (2.21)	0	7.00
Form constancy	3.57 (1.84)	0	7.56	3.64 (1.79)	0	7.50
Sequential memory	2.97 (2.03)	0	8.50	3.03 (1.84)	0	7.50
Figure-ground	2.92 (1.92)	0	7.63	2.90 (1.87)	0	6.69
Visual closure	3.97 (2.24)	0	8.50	3.96 (2.10)	0	7.50

### Individual Differences in TVPS-RDI and Quantitative ASD Traits

A regression equation including BAP-Q Total Average as well as FSIQ and Age was significant [*F*_(3, 83)_ = 18.14, *p* < 0.001, *R*^2^ = 0.40, Adj *R*^2^ = 0.37]. FSIQ, Age and BAP-Q Total Average were significant predictors of TVPS-FG RDI scores (*p*'s < 0.008). SRS Total raw score was not found to be a significant predictor of TVPS-FG RDI score (*p* < 0.69, NS). These results align with results reported above and demonstrate that within-task response variability in TVPS-FG can be predicted by quantitative ASD features as measured by the BAP-Q above and beyond the contribution of FSIQ and patient-level variables such as Age.

**Figure 4:**
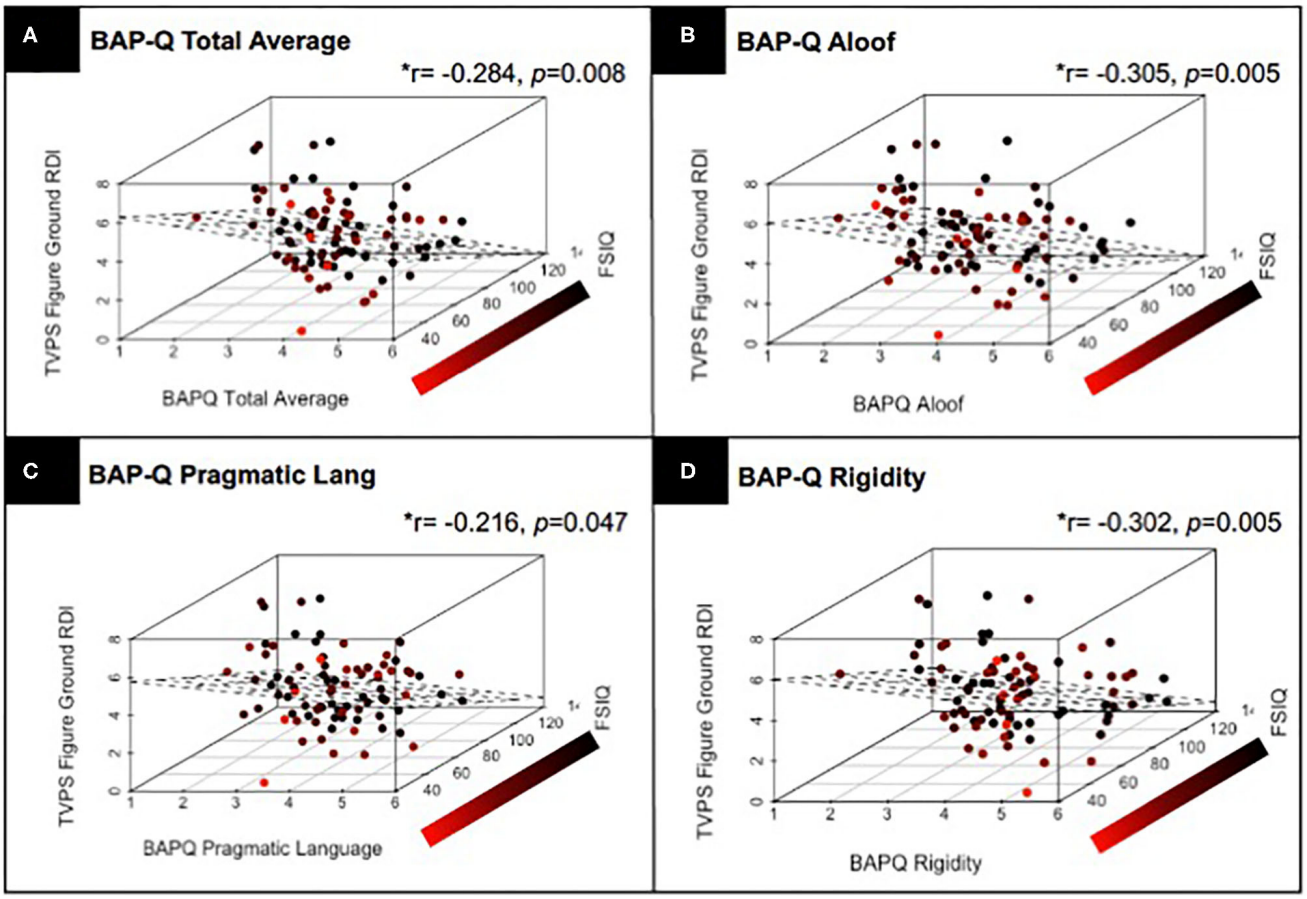
Results from a partial correlation (FSIQ) with correction for multiple comparisons, across children with and without ASD, demonstrating the relationship between TVPS-FG RDI scores and **(A)** BAP-Q Total scores; **(B)** BAP-Q Aloof subscales scores; **(C)** BAP-Q Pragmatic Language Scores; and **(D)** BAP-Q Rigidity subscale scores. Linear planes represent the results of a linear regression using FSIQ and **(A)** BAP-Q Total scores as well as **(B–D)** BAP-Q Aloof, Pragmatic Language, and Rigidity subscale scores to predict TVPS-FG subtest scores across children with and without ASD.

Next, we assessed the linear relationship between TVPS RDI subscale scores and quantitative ASD traits as assessed by the BAP-Q and SRS. Results from partial correlations, controlling for FSIQ and with correction for multiple comparisons indicated that TVPS-FG RDI scores were significantly related to BAP-Q Total (*p* = 0.008), BAP-Q Aloof (*p* = 0.005), BAP-Q Pragmatic Language (*p* = 0.047), and BAP-Q Rigidity (*p* = 0.005) (see Figure 4). Relationships were also noted between TVPS-FG RDI scores and SRS Total raw scores (*p* = 0.034), SCI (*p* = 0.040), RBRI (*p* = 0.030), Social Awareness (*p* = 0.009), and Social Communication (*p* = 0.048) subscale scores. There were a few significant relationships noted between other TVPS subscale scores and autism traits, as well. TVPS-VD RDI scores were found to be related to SRS Social Communication subscale scores (*p* = 0.040). TVPS-SR RDI scores were found to be related to BAP-Q Aloof subscale scores (*p* = 0.027). Finally, TVPS-SM RDI scores were found to be related to SRS Total raw scores (*p* = 0.046) as well as RBRI scores (*p* = 0.041). A summary of these results regarding the relationships between TVPS RDI scores and quantitative ASD traits can be found in Table 6.

**Table 6:** Partial correlation (FSIQ) between BAP-Q and TVPS RDI scores with correction for multiple comparisons.

	**VD RDI**	**VM RDI**	**SR RDI**	**FC RDI**	**SM RDI**	**FG RDI**	**VC RDI**
**SRS (raw scores)**							
Total	−0.210 *p = 0.053*	0.009 *p = 0.934*	−0.069 *p = 0.529*	−0.087 *p = 0.430*	–0.217^*^ ***p = 0.046***	–0.231^*^ ***p = 0.034***	−0.041 *p = 0.711*
SCI	−0.206 *p = 0.059*	−0.006 *p = 0.958*	−0.066 *p = 0.550*	−0.094 *p = 0.390*	−0.210 *p = 0.054*	–0.223^*^ ***p = 0.040***	−0.036 *p = 0.745*
RBRI	−0.206 *p = 0.058*	0.063 *p = 0.568*	−0.075 *p = 0.493*	−0.050 *p = 0.647*	−0.222^*^ ***p = 0.041***	−0.236^*^ ***p = 0.030***	−0.055 *p = 0.617*
Social awareness	−0.196 *p = 0.072*	0.043 *p = 0.697*	−0.059 *p = 0.594*	−0.167 *p = 0.127*	−0.199 *p = 0.067*	−0.282^**^ ***p = 0.009***	−0.059 *p = 0.589*
Social cognition	−0.143 *p = 0.192*	0.014 *p = 0.899*	−0.015 *p = 0.890*	−0.041 *p = 0.707*	−0.208 *p = 0.057*	−0.196 *p = 0.072*	−0.065 *p = 0.556*
Social communication	−0.223^*^ ***p = 0.040***	−0.019 *p = 0.861*	−0.055 *p = 0.617*	−0.116 *p = 0.291*	−0.195 *p = 0.073*	−0.215^*^ ***p = 0.048***	−0.043 *p = 0.698*
Social motivation	−0.191 *p = 0.080*	−0.020 *p = 0.853*	−0.117 *p = 0.286*	−0.032 *p = 0.769*	−0.199 *p = 0.067*	−0.182 *p = 0.095*	−0.040 *p = 0.716*
**BAP-Q**							
Total average	−0.157 *p = 0.150*	−0.015 *p = 0.889*	−0.139 *p = 0.206*	−0.104 *p = 0.346*	−0.135 *p = 0.217*	−0.284^**^ ***p = 0.008***	−0.052 *p = 0.635*
Aloof	−0.155 *p = 0.157*	−0.098 *p = 0.373*	−0.240^*^ ***p = 0.027***	−0.093 *p = 0.398*	−0.182 *p = 0.095*	−0.305^**^ ***p = 0.005***	−0.086 *p = 0.435*
Pragmatic language	−0.098 *p = 0.373*	0.010 *p = 0.998*	−0.022 *p = 0.842*	−0.109 *p = 0.323*	−0.146 *p = 0.182*	−0.216^*^ ***p = 0.047***	−0.033 *p = 0.764*
Rigid	−0.198 *p = 0.069*	0.046 *p = 0.675*	−0.101 *p = 0.360*	−0.146 *p = 0.182*	−0.131 *p = 0.231*	−0.302^**^ ***p = 0.005***	−0.059 *p = 0.594*

* *p < 0.05;*

** *p < 0.01. Bolded values are highlight significant results*.

### Item-Level Performance Across Groups With “High” and “Low” BAP Features

The proportion of correct vs. incorrect responses across TVPS-FG items was not found to be associated with higher BAP features (i.e., those with as compared to those without ASD) (*p*'s > 0.095, NS), with the exception of the fourth test item within the TVPS-FG subtest [*X*^2^_(1)_ = 3.982, *p* = 0.045]. Complete results from this analysis can be found in Supplement 6.

### Supplemental Analyses

Complete supplemental results described here are reported in Supplement 7. Briefly, we find that lower TVPS-FG RDI scores (*p* = 0.0271) were observed in the group with “high” BAP-Q Aloof subscale scores. This result indicates that we see a significant difference when groups are differentiated based on trait dimension cutoffs that mirror relationships observed in dimensional analyses with individual difference measurements of BAP features. Since this effect was not observed when groups were differentiated based on ASD clinical diagnosis, it emphasizes the complexity of these relationships and the value of including quantitative measurements of autism traits in such analyses.

Because it is known that as FSIQ decreases, autism symptoms increase, even in those without a clinical diagnosis of ASD (Kenworthy et al., 2010; Maxwell et al., 2013; DiCriscio and Troiani, 2018), it may be important to understand the interaction of FSIQ, autism traits, and visual perceptual abilities. To examine this, we also completed a supplemental analysis that defined groups based on “low” and “high” FSIQ (median split). Participants with “low” FSIQ also demonstrated lower TVPS-FG accuracy scores *but higher* TVPS-FG RDI scores (in contrast to *lower* TVPS-FG RDI scores in those with “high” BAP features). In Supplement 7, we present additional analyses to explore this potential interaction, but we do not identify significant effects of FSIQ x BAP-Q Aloof subscale scores, suggesting that the effect of IQ on TVPS-FG accuracy and response variability does not differ based on “high” and “low” ASD traits.

## Discussion

The current research aimed to assess visual-perceptual skills across the ASD phenotype using the TVPS-3, a standardized assessment of visual perception. We find that ASD traits are a significant predictor of TVPS performance in a pediatric cohort with a range of clinical features. Specifically, we find a significant association between performance on the Figure-Ground (FG) subtest of the TVPS and BAP-Q scores. No other subtest performance measures of the TVPS were found to be related to ASD traits. Thus, visual perceptual ability associated with the ASD phenotype appears to be specific to the TVPS FG subtest. We also report that item-level response dispersion on the TVPS is significantly associated with ASD traits, with lower TVPS RDI scores associated with increased ASD features.

Our findings contribute to the growing body of work on atypical visual perception in ASD and replicate and extend our previous work (DiCriscio and Troiani, 2017, 2018). To our knowledge, this is the largest study to date utilizing the TVPS in children with ASD. The TVPS-3 has been widely used to assess visual perception across various developmental populations (Hung et al., 1987; Hård et al., 2000, 2004; Davis et al., 2005) and has been highlighted as a reliable index of visual perceptual skills that would align with theories of atypical visual and sensory processing in ASD (Weak Central Coherence; Enhanced Perceptual Functioning; Happé, 1996; Mottron et al., 2006; Happé and Booth, 2008). Given the inconsistencies of research findings using the EFT and Navon figures (highlighted in the Introduction), future research on perceptual precedence and/or processing biases may benefit from using validated and comprehensive assessments like the TVPS.

A majority of the current research on domains of enhanced visual perception and ASD has been in adults (Almeida et al., 2010; Brock et al., 2011; Cribb et al., 2016). Those studies which have dimensionally assessed *subclinical* ASD features tend to dichotomize groups of individuals that lack neurodevelopmental diagnoses based on high- vs. low-ASD trait load (Cribb et al., 2016). Our reported results highlight the utility of dimensional assessment of ASD traits across a heterogeneous pediatric cohort that includes those with other neurodevelopmental concerns.

In the current study and in our previous research (DiCriscio and Troiani, 2018), BAP-Q scores were a significant predictor of TVPS-FG performance, while SRS scores were not. It is important to note that there are differences in subjective content in the BAP-Q and SRS. The SRS is widely known as a clinical research assessment of ASD features and focuses on the capture of relative deficits in social behaviors that would indicate clinically significant impairments associated with a diagnosis of ASD above a predetermined threshold. In contrast, the BAP-Q was created to quantify *subclinical* traits across core symptom domains of the autism phenotype. The BAP-Q includes items with similar content; but more broadly focuses on the characterization of subclinical psychosocial as well as other behavioral features of ASD (i.e., restricted and repetitive behaviors as well as sensory processing features). Our results with BAP-Q only may be due to differences in the measurement of social abilities and the subjective content of test items in the SRS and BAP-Q questions. For example, the SRS includes questions focused on applied social skills (i.e., appropriately ending conversation, awareness of others' thoughts or feelings, monitoring facial expressions). Items from the BAP-Q Aloof subscale are focused on the rewarding or motivating aspect of social behavior or social interaction (i.e., “I like being around other people”). Thus, it remains unclear how visual perceptual differences in ASD may be associated with core symptoms that may be measured differently using various surveys and assessments used for research and clinical screening.

Previous work by Falkmer et al. (2016) found no differences in TVPS-FG performance in a small sample of individuals with and without high functioning ASD (described as Aspergers in the publication). Our results are similar to those reported in Falkmer et al. (2016), with similar task accuracy between ASD and non-ASD samples, even though our non-ASD sample was also enriched for traits associated with atypical development. We did find that our ASD subsample demonstrated *poorer* task performance on the two subtests that involve a memory component and therefore may be considered more cognitively demanding relative to the other five TVPS subtests. Thus, those with ASD and more profound cognitive impairments may have found these subtests exceptionally challenging due to the increased task demands. The absence of overall differences between groups in the current study and previous work by Falkmer et al. (2016) emphasizes that visual perceptual differences are not necessarily specific to an ASD diagnosis and further highlight the need to dimensionally assess ASD traits to uncover trait domains that relate to visual perceptual anomalies across the neurodevelopmental spectrum.

A novel aspect of the current study was the item-level analysis and quantitative assessment of within-task variability across the TVPS in children with and without ASD. While previous studies have utilized item-based analyses on psychometric and cognitive data in ASD (Hallenbeck et al., 1965), the current research presents a novel use of an RDI score as an objective index of response pattern profile in the context of visual perception. The modified RDI metric reported here captures item response variability (i.e., incorrect responses) in cognitive and behavioral assessments where test item difficulty may progress across test administration, with lower scores indicating a more consistent and proficient response pattern. In the current context, those with lower RDI scores are interpreted to reflect more consistent item responses in a manner that aligns with item difficulty. We do not find significant differences in TVPS RDI scores between ASD and non-ASD groups; however, our results demonstrated individual differences in TVPS-FG RDI scores that scaled with the presence of quantitative ASD traits. Previous research using RDI in item-level analysis has reported *increased* RDI (interpreted as *diminished efficiency*) associated with language processing abilities in those with ASD (Hare-Harris et al., 2019). In the current study, we report an inverse relationship between RDI and visual perceptual skills. Interpreting the RDI in the context of previous work, this metric may indicate *increased efficiency* in cognitive/perceptual processes associated with ASD traits when the demand is placed on localizing features over extracting a more global or gestalt whole. The RDI metric utilized here and in previous studies (VanMeter et al., 1997; Hare-Harris et al., 2019) underscores the utility of characterizing response pattern profiles at the individual item-level. However, it remains unclear whether visual perceptual differences in ASD are tied to an enhanced perceptual processing style or “state” as opposed to traits that scale with the presence of ASD features.

The RDI as reported here does not assess specific items or item characteristics that may distinguish populations with a clinical diagnosis of ASD from those without. We did not find a significant association between correct vs. incorrect responses and higher as compared to lower autism traits at the item-level with the exception of one test item (see Supplement 6 for these results). The RDI is not intended to be used as a form of discriminant item analysis but rather quantifies the distribution of response variability across individuals. A current challenge in vision and perceptual research within ASD is that variable task-demands and stimulus sets influence the reported direction and interpretation of results. Research in other cognitive domains impacted in ASD has suggested that future studies in those with and without diagnostic features of ASD and overlapping neurodevelopmental syndromes requires in-depth knowledge of the psychometric properties of the assessment or task and whether item-level response profiles can be equated across groups (Facon et al., 2011; McKenzie et al., 2020). The use of a standardized assessment such as the TVPS is a strength of the current research; however, additional research is necessary to objectively assess components that may be best able to distinguish individuals based on cognitive or neurodevelopmental features, such as variance of low-level visual properties across test items (Bertone et al., 2005; Ashwin et al., 2009; Milne and Szczerbinski, 2009; Milne et al., 2009).

There are additional limitations in the current study that should be addressed in future research. First, we collected and coded data according to standardized ceiling/stop rules as outlined by the TVPS scoring manual. Future investigations would benefit from administering all TVPS test items in order to assess the predictive validity across items as well as possible features of individual test items (i.e., cognitive demands, low-level visual properties) that may discriminate individuals with ASD from healthy controls and/or those with other neurodevelopmental conditions. Furthermore, we acknowledge that other standardized cognitive and perceptual assessments and specific subtests have been used in behavioral research studies of individuals with and without ASD (Jolliffe and Baron-Cohen, 1997; Barron-Linnankoski et al., 2015; Green et al., 2016) and in other pediatric populations (Ortibus et al., 2011); however, it is important to note the scope of several of these measures is on visuo-motor skill and/or motion perception as opposed to visual perception and/or perceptual organization including low-level visual processing such as those assessed as a part of the TVPS. Additionally, some of the above-mentioned assessments emphasize outcome measures which identify and “label” impairment or deficits rather than dimensionally characterizing individuals in overall skill. Despite these differences in the subjective content and scope of cognitive and perceptual batteries, future studies should continue to explore the use of the TVPS and other standardized assessments of cognitive, visual and perceptual processes that focus on the intersection of these domains (i.e., perception, biological motion, visuo-motor development and clinical features of ASD). Finally, we also acknowledge that our ASD subsample was primarily comprised of males and that the male: female ratios between those participants with and without ASD was significantly different (see Table 1). Additionally, our included quantitative measures of ASD traits included the BAP-Q and previous research has reported different distributions of ASD traits and BAP-Q scores between males and females (Hurley et al., 2007). It will be important for future research to improve identification and diagnosis of ASD in females in order to better understand whether the relationship between autism traits and visual perception in females is similar to those observed in males. Current diagnostic measures are biased toward detecting ASD features in males and thus do not capture the full distribution of social impairments with sufficient specificity to distinguish sex differences in the subtle presentation of symptoms (Ehlers et al., 1999; Rutter et al., 2003). Recent estimates suggest a male: female ratio of at least 3:1–4:1 within ASD (Brugha et al., 2011; Kirkovski et al., 2013; Halladay et al., 2015; Loomes et al., 2017); however, studies of cohorts with a genetic etiology associated with an ASD diagnosis have reported ratios closer to 1:1 (Jacquemont et al., 2014). Thus, it may be informative for future work to assess visual perceptual skills and their relationship to autism traits within populations that have ASD risk with more similar prevalence in males and females, such as those with specific genetic etiologies.

Despite these limitations, the current research makes a significant contribution to the growing body of research on visual perception in ASD. Taken together, these results emphasize the need for the quantitative and dimensional assessment of ASD traits and may explain why findings regarding visual perception in ASD are mixed, as they may be influenced by the trait distribution of a given sample of participants. Further, this work also provides normative TVPS-3 data on all 7 subtests in the largest ASD cohort to date and lays the groundwork for future research describing areas of perceptual skill vs. impairment in neurodevelopmental populations.

## Data Availability Statement

The raw data supporting the conclusions of this article will be made available by the authors, without undue reservation.

## Ethics Statement

The studies involving human participants were reviewed and approved by Geisinger Institutional Review Board. Written informed consent to participate in this study was provided by the participants' legal guardian/next of kin.

## Author Contributions

AD and VT designed the research, interpreted the data, and critically revised the manuscript. AD collected the data and drafted the manuscript. AD analyzed the data with guidance from VT and assistance from JS. All authors have read, approved the final version of the manuscript, and reviewed the manuscript.

## Conflict of Interest

The authors declare that the research was conducted in the absence of any commercial or financial relationships that could be construed as a potential conflict of interest.
