# Supplementary material for: Comprehensive Assessment of Visual Perceptual Skills in Autism Spectrum Disorder
Source: Front Psychol. 2021 Jul 13;12:662808. doi: 10.3389/fpsyg.2021.662808 (PMC8314997; doi:10.3389/fpsyg.2021.662808)

Supplement 1.

Behavioral and parent report measures described in the main text were normally distributed according to a Shapiro-Wilks test of normality (p’s>0.06) with the exception of scores on TVPS-Figure Ground subscale (*p*=0.023), BAP-Q Aloof (*p*=0.011), and BAP-Q Rigidity (*p*=0.044). TVPS RDI scores also deviated from a normal distribution (*p*’s<0.21), with the exception of the TVPS-FC subtest (*p*>0.1714, NS). Thus, a nonparametric, Mann-Whitney *U* test was used to assess differences in demographic variables as well scores on behavioral measures between children with and without ASD in the current study (i.e. ASD versus non-ASD). Our subsample of children with ASD did not differ in age from those children without ASD (*U*= 1148.5, p=0.876); however, children with ASD did have lower FSIQs as compared to children without ASD (*U*=500.0, *p*<0.001). Additionally, children with ASD demonstrated higher BAP-Q Total scores (*U*= 236.0, p<0.001), SRS Total raw scores (*U*=362.5, p<0.001), and higher scores across all BAP-Q and SRS subscales (p’s<0.001) as compared to children without ASD.

Age was not found to be associated with BAP-Q scores (*p*’s>0.31, NS), SRS scores (*p*’s>0.16, NS), or TVPS scores (*p*’s>0.15, NS) with the exception of the TVPS Visual Closure subtest (r= 0.284, *p*=0.007). FSIQ was found to be related to TVPS Overall scores (r= 0.636, *p*<0.001), all TVPS subtest scores (*p*’s<0.001), BAP-Q Total score (r= -0.295, *p*=0.004) and BAP-Q subscale scores (p’s<0.009) with the exception of the Aloof subscale (*p*>0.142). FSIQ was also found to be related to SRS Total raw score (r= -0.278, *p*=0.007) as well as all subscale scores (*p*’s<0.034). As we have reported here and previous research has also demonstrated (DiCriscio and Troiani 2018; Kenworthy et al. 2010; Maxwell et al. 2013), FSIQ was found to be related to our measures of individual differences in autism traits, as well as visual perceptual performance on the TVPS. We also note that RDI scores for all TVPS subtests were found to be significantly related to TVPS performance (p’s<0.001), indicating those with a lower RDI index had higher TVPS scores (increased performance). We account for this multi-collinearity in our subsequent analyses reported in the main text. Finally, a partial correlation controlling for age as well as FSIQ was run to determine the internal concurrent validity of the BAP-Q and SRS within this heterogeneous cohort. BAP-Q Total score was found to be related to SRS Total raw score (r= 0.668, *p*<0.001). All BAP-Q and SRS subscales were also found to related (*p*’s<0.001). See table below for complete results.


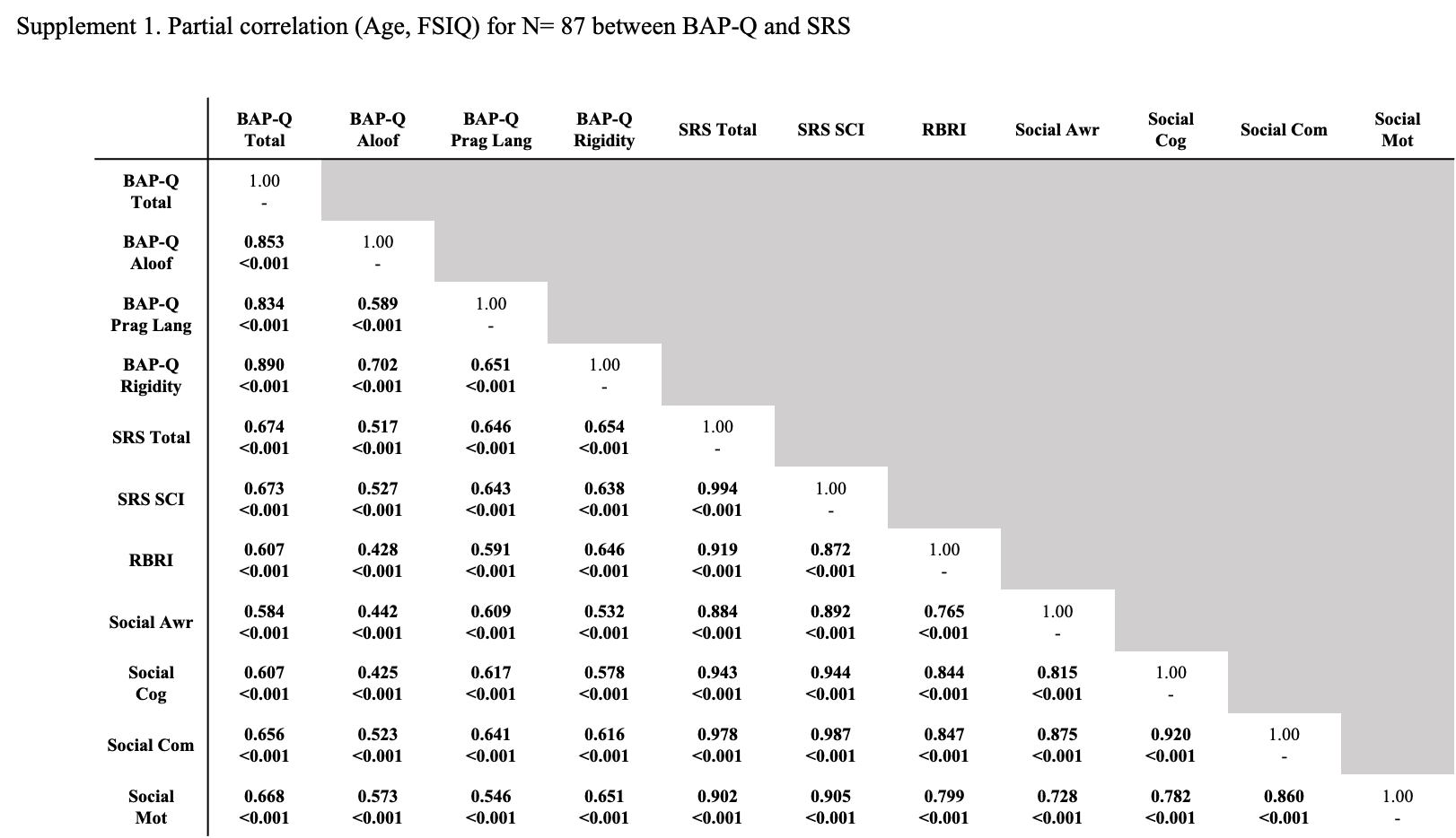

Supplement: Supplementary file 1 [file Data_Sheet_1.docx]
