# Supplementary figures and images for: Comprehensive Assessment of Visual Perceptual Skills in Autism Spectrum Disorder
Source: Front Psychol. 2021 Jul 13;12:662808. doi: 10.3389/fpsyg.2021.662808 (PMC8314997; doi:10.3389/fpsyg.2021.662808)

Supplement 2- TVPS Index Standard Scores

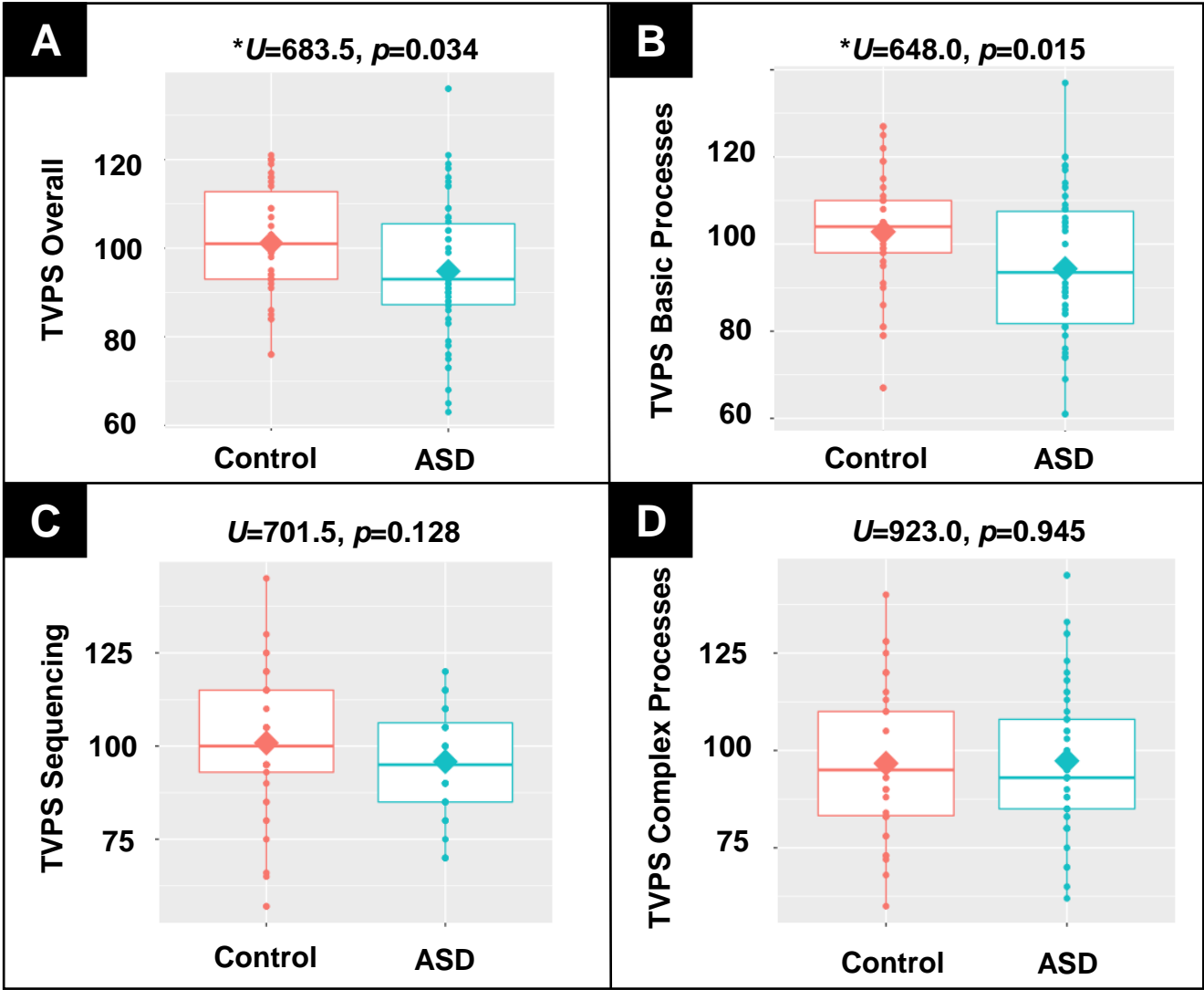

Supplement: Supplementary file 2 [file Data_Sheet_2.pdf]

Supplement 3- TVPS Subtest Scaled Scores

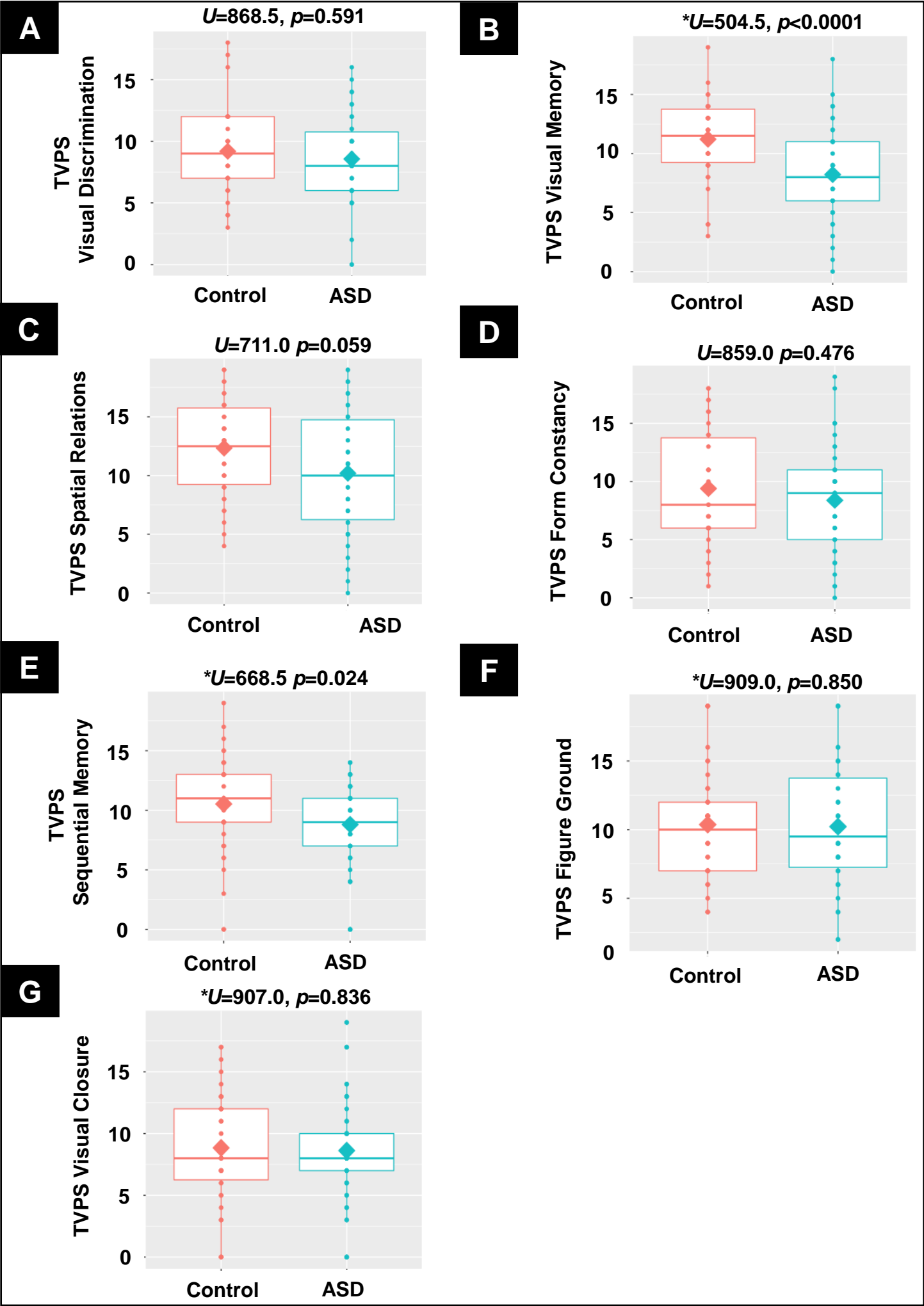

Supplement: Supplementary file 3 [file Data_Sheet_3.pdf]
