# Supplementary material for: Comprehensive Assessment of Visual Perceptual Skills in Autism Spectrum Disorder
Source: Front Psychol. 2021 Jul 13;12:662808. doi: 10.3389/fpsyg.2021.662808 (PMC8314997; doi:10.3389/fpsyg.2021.662808)
Supplement: Supplementary file 4 [file Data_Sheet_4.pdf]

Supplement 4. Partial correlation (Age, FSIQ) for N= 87 between TVPS and BAP-Q

		BAP-Q				TVPS Subtests						
		Total Average	Aloof	Prag Lang	Rigidity	VD	VM	SR	FC	SM	FG	VC
BAP-Q	Total Average	1.00<br>-										
	Aloof	0.850**<br><0.001	1.00<br>-									
	Prag Lang	0.840**<br><0.001	0.580**<br><0.001	1.00<br>-								
	Rigidity	0.897**<br><0.001	0.710**<br><0.001	0.684**<br><0.001	1.00<br>-							
TVPS Subtests	VD	0.125<br>0.253	0.092<br>0.402	0.141<br>0.198	0.139<br>0.204	1.00<br>-						
	VM	-0.102<br>0.353	-0.035<br>0.753	-0.092<br>0.403	-0.177<br>0.106	0.332**<br>0.002	1.00<br>-					
	SR	0.043<br>0.695	0.058<br>0.600	0.064<br>0.560	-0.009<br>0.936	0.333**<br>0.002	0.195<br>0.073	1.00<br>-				
	FC	0.097<br>0.379	-0.001<br>0.989	0.131<br>0.232	0.129<br>0.239	0.316**<br>0.003	0.160<br>0.144	0.156<br>0.153	1.00<br>-			
	SM	-0.022<br>0.844	-0.014<br>0.896	0.093<br>0.396	-0.061<br>0.580	0.191<br>0.080	0.247*<br>0.023	0.339**<br>0.002	0.288**<br>0.007	1.00<br>-		
	FG	0.314**<br>0.003	0.266*<br>0.014	0.316**<br>0.003	0.305**<br>0.005	0.520**<br><0.001	0.264*<br>0.014	0.338**<br>0.002	0.317**<br>0.003	0.396**<br><0.001	1.00<br>-	
	VC	0.051<br>0.644	0.046<br>0.677	0.138<br>0.209	0.002<br>0.987	0.486**<br><0.001	0.097<br>0.379	0.213<br>0.051	0.273*<br>0.012	0.276*<br>0.011	0.498<br><0.001	1.00<br>-
