# Supplementary material for: Comprehensive Assessment of Visual Perceptual Skills in Autism Spectrum Disorder
Source: Front Psychol. 2021 Jul 13;12:662808. doi: 10.3389/fpsyg.2021.662808 (PMC8314997; doi:10.3389/fpsyg.2021.662808)
Supplement: Supplementary file 5 [file Data_Sheet_5.docx]

Supplement 5.


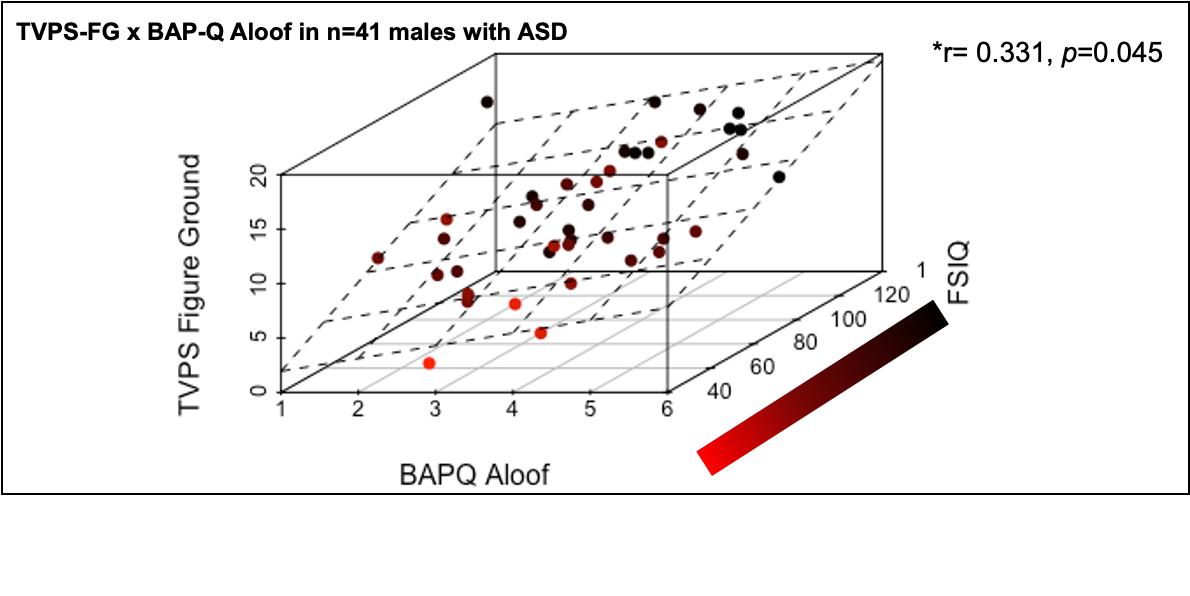


Due to the disproportionate number of males as compared to females in our sample (i.e. only n=7 females with ASD were included) and results from previous research, we repeated our analyses in male participants with and without ASD. We first explored possible group differences in TVPS performance between males with ASD as compared to those without ASD. Male participants with and without ASD did not demonstrate differences in TVPS performance across TVPS subtests (p’s>0.082, NS), with the exception of the TVPS-SM subtest (*p*=0.014). Male participants without ASD scored higher on the TVPS-SM subtest (mean= 11.79 ± 3.64) as compared to male participants with ASD (mean=8.78 ± 3.13).

We repeated our correlation based analyses described in the main text and included only males with an ASD diagnosis (n=41). Results from a partial correlation, controlling for age and FSIQ with correction for multiple comparisons, indicated a significant relationship between the TVPS-FG subtest and BAP-Q Aloof subscale scores (r= 0.331, *p*=0.045). See figure above. We do not wish to present these results as meaningful gender differences in visual perceptual skill and the presence of ASD traits in male as compared to females, as our sample in the current study left us underpowered to explore the same relationship in females with ASD. However, these results align with our previously published work reporting a specific relationship between performance on the TVPS-FG and ASD traits as measured by the BAP-Q Aloof subscale in healthy adult males (DiCriscio and Troiani, 2017). Finally, we also repeated analyses and included *all individuals with ASD* (male and female). Results from a partial correlation between TVPS and BAP-Q (controlling for FSIQ) did not indicate significant relationships between any of the TVPS subtests and BAP-Q Total average score (*p*’s>0.24, NS) nor the BAP-Q subscale scores (*p*’s>0.15, NS).
