# Supplementary material for: Comprehensive Assessment of Visual Perceptual Skills in Autism Spectrum Disorder
Source: Front Psychol. 2021 Jul 13;12:662808. doi: 10.3389/fpsyg.2021.662808 (PMC8314997; doi:10.3389/fpsyg.2021.662808)
Supplement: Supplementary file 6 [file Data_Sheet_6.docx]

| **TVPS-FG Item #1**, $X$^2^(1)= 0.0004, *p*=0.998 | | | |
| --- | --- | --- | --- |
|  |  | **Accuracy** | |
|  |  | **‘0’** | **‘1’** |
| **BAP-Q Total** | **Low** | 1 | 43 |
|  | **High** | 2 | 41 |

| **TVPS-FG Item #2,** $X$^2^(1)= 0.107, *p*=0.734 | | | |
| --- | --- | --- | --- |
|  |  | **Accuracy** | |
|  |  | **‘0’** | **‘1’** |
| **BAP-Q Total** | **Low** | 7 | 37 |
|  | **High** | 9 | 34 |

| **TVPS-FG Item #3,** $X$^2^(1)= 1.287, *p*= 0.257 | | | |
| --- | --- | --- | --- |
|  |  | **Accuracy** | |
|  |  | **‘0’** | **‘1’** |
| **BAP-Q Total** | **Low** | 6 | 38 |
|  | **High** | 11 | 32 |

| **TVPS-FG Item #4,** $\boldsymbol{X}$**^2^(1)= 3.982, *p*=0.045*** | | | |
| --- | --- | --- | --- |
|  |  | **Accuracy** | |
|  |  | **‘0’** | **‘1’** |
| **BAP-Q Total** | **Low** | 11 | 33 |
|  | **High** | 3 | 40 |

| **TVPS-FG Item #5,** $X$^2^(1)= 0.008, *p*=0.929 | | | |
| --- | --- | --- | --- |
|  |  | **Accuracy** | |
|  |  | **‘0’** | **‘1’** |
| **BAP-Q Total** | **Low** | 17 | 27 |
|  | **High** | 18 | 25 |

| **TVPS-FG Item #6,** $X$^2^(1)= 0.001, *p*=0.998 | | | |
| --- | --- | --- | --- |
|  |  | **Accuracy** | |
|  |  | **‘0’** | **‘1’** |
| **BAP-Q Total** | **Low** | 12 | 32 |
|  | **High** | 11 | 32 |

| **TVPS-FG Item #7,** $X$^2^(1)= 0.000, *p*=0.999 | | | |
| --- | --- | --- | --- |
|  |  | **Accuracy** | |
|  |  | **‘0’** | **‘1’** |
| **BAP-Q Total** | **Low** | 22 | 22 |
|  | **High** | 21 | 22 |

| **TVPS-FG Item #8,** $X$^2^(1)= 0.000, *p*=0.999 | | | |
| --- | --- | --- | --- |
|  |  | **Accuracy** | |
|  |  | **‘0’** | **‘1’** |
| **BAP-Q Total** | **Low** | 21 | 23 |
|  | **High** | 20 | 23 |

| **TVPS-FG Item #9,** $X$^2^(1)= 0.104, *p*=0.746 | | | |
| --- | --- | --- | --- |
|  |  | **Accuracy** | |
|  |  | **‘0’** | **‘1’** |
| **BAP-Q Total** | **Low** | 23 | 21 |
|  | **High** | 20 | 23 |

| **TVPS-FG Item #10,** $X$^2^(1)= 0.005, *p*=0.942 | | | |
| --- | --- | --- | --- |
|  |  | **Accuracy** | |
|  |  | **‘0’** | **‘1’** |
| **BAP-Q Total** | **Low** | 31 | 13 |
|  | **High** | 29 | 14 |

| **TVPS-FG Item #11,** $X$^2^(1)= 0.006, *p*=0.936 | | | |
| --- | --- | --- | --- |
|  |  | **Accuracy** | |
|  |  | **‘0’** | **‘1’** |
| **BAP-Q Total** | **Low** | 29 | 15 |
|  | **High** | 27 | 16 |

| **TVPS-FG Item #12,** $X$^2^(1)= 0.018, p=0.894 | | | |
| --- | --- | --- | --- |
|  |  | **Accuracy** | |
|  |  | **‘0’** | **‘1’** |
| **BAP-Q Total** | **Low** | 26 | 18 |
|  | **High** | 27 | 16 |

| **TVPS-FG Item #13,** $X$^2^(1)= 0.000, *p*=0.999 | | | |
| --- | --- | --- | --- |
|  |  | **Accuracy** | |
|  |  | **‘0’** | **‘1’** |
| **BAP-Q Total** | **Low** | 34 | 10 |
|  | **High** | 33 | 10 |

| **TVPS-FG Item #14**, $X$^2^(1)= 2.082, *p*=0.149 | | | |
| --- | --- | --- | --- |
|  |  | **Accuracy** | |
|  |  | **‘0’** | **‘1’** |
| **BAP-Q Total** | **Low** | 30 | 14 |
|  | **High** | 36 | 7 |

| **TVPS-FG Item #15**, $X$^2^(1)= 0.178, *p*=0.673 | | | |
| --- | --- | --- | --- |
|  |  | **Accuracy** | |
|  |  | **‘0’** | **‘1’** |
| **BAP-Q Total** | **Low** | 31 | 13 |
|  | **High** | 33 | 10 |

| **TVPS-FG Item #16,** $X$^2^(1)= 0.000, *p*=0.999 | | | |
| --- | --- | --- | --- |
|  |  | **Accuracy** | |
|  |  | **‘0’** | **‘1’** |
| **BAP-Q Total** | **Low** | 34 | 10 |
|  | **High** | 34 | 9 |
