# Supplementary material for: Comprehensive Assessment of Visual Perceptual Skills in Autism Spectrum Disorder
Source: Front Psychol. 2021 Jul 13;12:662808. doi: 10.3389/fpsyg.2021.662808 (PMC8314997; doi:10.3389/fpsyg.2021.662808)

Supplement 7.

The analysis and results reported in the main manuscript place specific emphasis on quantitatively characterizing individual differences in perceptual metrics and clinically relevant traits as compared to more common approaches in behavioral research that draw on group comparisons. Given the significant relationships reported across FSIQ, quantitative measures of ASD, and our TVPS metrics of performance and item-level response variability, we further assessed the effects of IQ and ASD traits on TVPS-FG accuracy and TVPS-FG RDI scores between children with ‘lower’ as compared to ‘higher’ IQ and BAP-Q Total and subscale scores determined by a median split. Our TVPS-FG metrics deviated from a normal distribution (*p*’s< 0.02), thus nonparametric tests with correction for multiple comparisons were used to assess differences in TVPS accuracy and response variability between those with “higher” as compared to “lower” FSIQ and ASD traits. Results indicated that participants with ‘lower’ FSIQ demonstrated lower TVPS-FG accuracy scores (*p*=0.0005) and higher TVPS-FG RDI scores (*p*=0.001). There were no significant differences in TVPS-FG accuracy and RDI scores between individuals with ‘higher’ as compared to ‘lower’ BAP-Q total and subscale scores (*p*’s<0.792, NS), with the exception of differences in TVPS-FG RDI scores between those with ‘higher’ versus lower’ BAP-Q Aloof subscale scores. Lower TVPS-FG RDI scores (*p*= 0.03) were observed in those with higher BAP-Q Aloof subscale scores. See figure below. Finally, given results from group comparisons, nonparametric permutational analysis of variance (PERMANOVA) was used to explore the possible interaction effects of FSIQ x BAP-Q Aloof. Results from PERMANOVA confirmed a main effect of both FSIQ and BAP-Q Aloof subscale scores *(p’s*<0.002); however, a significant interaction effect of FSIQ and BAP-Q Aloof was not found to be significant (*p*=0.932, NS).

Results from this supplemental analysis suggests that the effect of IQ on TVPS-FG accuracy and response variability does not differ based on ‘higher’ or ‘lower’ ASD traits and underscores the utility of a quantitative approach as was described in the main text of the manuscript. Group comparisons between diagnostic groups and/or groups established by arbitrary cutoffs as is outlined here (i.e. ‘lower’ vs ‘higher’), overlook meaningful variability and individual differences in cognitive and perceptual traits that may scale with the presence of core clinical features of ASD. As outlined in the Discussion section, continued work in larger samples is necessary in order to comprehensively characterize the utility of standardized measures of visual perception such as the TVPS in identifying clinically meaningful neurodevelopmental phenotypes and possibly sub-phenotypes across cognitive and behavioral domains.


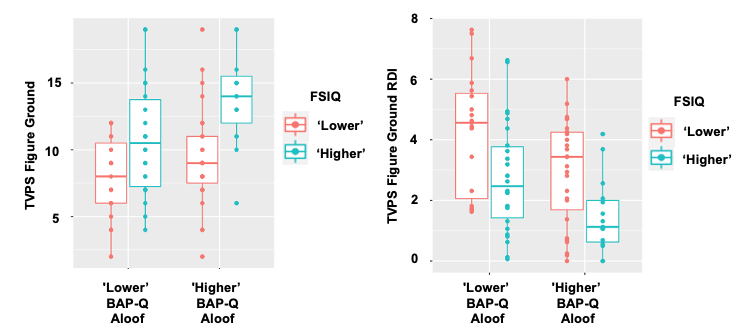

Supplement: Supplementary file 7 [file Data_Sheet_7.docx]
